# Two Concepts of Belief Strength: Epistemic Confidence and Identity Centrality

**DOI:** 10.3389/fpsyg.2022.939949

**Published:** 2022-06-29

**Authors:** Neil Van Leeuwen

**Affiliations:** Department of Philosophy, Neuroscience Institute, Georgia State University, Atlanta, GA, United States

**Keywords:** belief, credence, measurement, identity, epistemic confidence, identity centrality

## Introduction: a Difference Worth Measuring

What does it mean to have “strong beliefs”? My thesis is that it can mean two very different things. That is, there are two distinct psychological features to which “strong belief” can refer, and these often come apart. I call the first feature *epistemic confidence* and the second *identity centrality*. They are conceptually distinct and, if we take ethnographies of religion seriously, distinct in fact as well. If that's true, it's methodologically important for the psychological sciences to have *measures* that tease them apart.

## Epistemic Confidence vs. Identity Centrality

The following hypothetical case illustrates the distinction.

Johan (a young Afrikaner man) frequently insists that his deceased father was an opponent of Apartheid in the 1970s and 1980s. This is part of the standard narrative of his family history he gives to people he meets. Yet privately he knows he doesn't have that much evidence it's true, and sometimes he suspects his father just told him that to make himself look good.
Johan also has a great deal of certain knowledge of various things that matter little to him. He knows Istanbul used to be called Constantinople, one is technically not a prime, and Toyota has manufacturing facilities in South Africa.

In this case, identity centrality and epistemic confidence come apart in both directions. Johan has a *high* degree of identity centrality for the idea that his father opposed Apartheid (that idea is part of his social identity), but he has a *low* degree of epistemic confidence in that idea (he's not sure it's true). Conversely, he has a *high* degree of epistemic confidence that Istanbul used to be called Constantinople, but that same idea, for him, has a *low* degree of identity centrality—if it has any.

The difference between the two psychological features is also apparent in real-world ethnographies of religion. I focus here on ethnographies of The Vineyard Church (a large, representative American Evangelical sect^1^) by Luhrmann (2012) and Bialecki (2017). A central practice of the Vineyard is “hearing” the voice of God, where this typically involves having internal auditory mental imagery. Yet Vineyard members often speak of such experiences like this: “Sometimes when we think it's the spirit moving, it's just our burrito from lunch” and “There's always a choice to believe what it is” (Luhrmann, 2012, p. 70). Relatedly, Bialecki notes that Vineyard members commonly joke about the difficulty of determining whether the feelings they're having are from God or from the pizza they had for lunch. Hence, Vineyard members are often *unsure* (epistemically unconfident) that God spoke to them. Uncertainty is apparent also in that they describe it as a “choice” to believe and commonly find it *difficult* to “believe,” as do members^1^ of other Christian sects (Appiah, 2019, p. 37–38). Vineyard members often struggle with *doubt*. One Vineyard member even said, “I don't believe it. But I'm sticking with it. That's my definition of faith” (Luhrmann, 2012, p. 316). I propose that this cluster of phenomena can best be explained by positing that many Vineyard members have a low degree of epistemic confidence in their “beliefs,” while those “beliefs” are nevertheless central to their identities, which is why they keep showing up, putting in effort, and saying things like “I'm sticking with it.” Without being confident that their “beliefs” describe how things really are, they maintain them because of who *they* are (cf. Heiphetz et al., 2014).

To capture the difference in question, let the following serve as working definitions that can be refined through iterative stages of empirical inquiry and theoretical reflection.

Epistemic confidence: The degree to which someone feels a belief state approximates *knowledge*.

(Knowledge, for purposes of this definition, implies clear contents, objective truth, and rational justification^2^.)

Identity centrality: The degree to which someone experiences a belief state as part of their *social identity*.

(Social identity, for purposes of this definition, is a cluster of psychological states and behavioral dispositions that constitute someone as a member of an actual or potential in-group, or that an individual uses to achieve a desired social position^3^.)

To be clear, I am not suggesting that identity centrality is more important than epistemic confidence, or *vice versa*. They are just different psychological features that should not be confused. So now let's examine how some current measures of “belief” in psychology of religion fare in light of this distinction^4^.

On Fullerton and Hunsberger's (1982) “Christian Orthodoxy Scale,” respondents write down integers ranging from −3 (“strongly disagree”) to +3 (“strongly agree”) next to various claims. For example: “God exists as: Father, Son, and Holy Spirit,” “Man is *not* a special creature made in the image of God, he is simply a recent development in the process of animal evolution” (where this is *contrary* to Christian orthodoxy), “Jesus Christ was the divine Son of God,” etc.

No doubt the Fullerton and Hunsberger scale captures something important about religious psychology in that it measures Christian orthodoxy in some sense. But suppose a researcher administered the scale to a group of participants and most of them put “+3” next to all orthodox items and “−3” next to all contra-orthodox items. Would that researcher know whether the “+3” and “−3” responses were driven more by *epistemic confidence* or more by *identity centrality*? She would not. The reason why is that *either* psychological feature could cause a participant to put down “+3” next to the orthodox items. People who are epistemically confident an idea is true will typically be motivated to express “strong agreement” with it (conversely for disagreement), but so will people for whom that idea is central to their identity. So the Fullerton and Hunsberger scale doesn't capture this important difference.

To put the point abstractly, for any proposition *p*, a person with a high degree of epistemic confidence that *p* and a person with a high degree of identity centrality for *p* are both likely to put “+3” next to a sentence expressing *p*. So the scale does not discriminate.

Furthermore, if we trust the ethnographies just mentioned, this is a domain in which we might expect the two features to come apart. A researcher might hypothesize that many orthodox Christians are high in identity centrality with respect to orthodox beliefs, while being low or lower in epistemic confidence. The scale itself, however, would not help *test* that hypothesis.

This is not the place for an exhaustive catalog of belief measures, but it is worth observing how some prominent measures tilt toward one psychological feature or another, while others are entirely ambiguous between them.

In developmental psychology, Paul Harris has initiated a cross-cultural research program that compares people's confidence (probed in various ways) in the existence of scientific entities (e.g., germs, oxygen, etc.) to their confidence in the existence of supernatural entities of their religions (God, angels, etc.). Findings indicate that, even in religious societies like the United States and Iran, children and adults alike generally have lower degrees of confidence in religious than in scientific entities (Harris et al., 2006; Davoodi et al., 2018; Clegg et al., 2019). Measurement instruments in this line of research tilt in the direction of tracking epistemic confidence, but it is hard to rule out that identity centrality is also playing a role in driving some of the “confident” responses concerning religious entities. Hence, the findings could *understate* people's difference in epistemic confidence concerning scientific and religious entities.

Within social psychology of religion, some measures do help track identity centrality. Lindeman et al. (2020), for example, have items that probe how desirable religiosity is for respondents, whether they take religion to be harmful, and the degree of strong emotions elicited by religion. They also ask directly: “How important are religious attitudes to your identity?” Such questions are indeed useful in tracking the identity centrality. But they do not offer much in terms of assessing whether and how epistemic confidence and identity centrality converge or diverge. They also do not assess those psychological features in relation to *specific* religious doctrines and stories, such as the existence of a triune God or the Virgin Birth.

In psychology of religion more generally, there are indeed measures that track adherence to specific belief contents, but (like Fullerton and Hunsberger) without distinguishing epistemic confidence and identity centrality. Jong et al. (2013), for example, include these items: “There exists an all-powerful, all-knowing, loving God,” “Some people will go to Heaven when they die,” etc. Their scale runs from −4 (strongly disagree) to +4 (strongly agree). They write, “The two ends of the scale are therefore designed to indicate extreme disbelief or atheism... and confident belief... whereas the midpoint of the scale (i.e., 0) implies agnosticism or uncertainty” (496). Other measures of “belief” have similar Likert scales (Tobacyk, 2004; Pennycook et al., 2012; e.g., Lindeman et al., 2015). The “strongly” in these scales is what's problematic: strong in *which way*—epistemic confidence or identity centrality? We don't know.

## Discussion: Where Do We Go From Here?

But why, one might ask, should we *want* measures that separate those features? The answer is that the difference matters for both descriptive psychological research and for normative philosophical research.

With regards to descriptive psychology, I submit that epistemic confidence and identity centrality are likely to differ along the following practically important dimensions: formation conditions (how a given attitude is formed), extinction conditions (how a given attitude is extinguished), and action guidance (what sorts of behaviors that attitude generates and how). In other words, an epistemically confident belief that *p* is likely to be formed, be revised, and generate action differently from a belief that *p* that is central to one's identity.

The table above (Table 1) lays out likely differences. (Here, the properties of epistemic confidence are standard in literature on “degrees of belief” in decision theory and formal epistemology^5^.)

**Table 1:** Dimensions of difference: epistemic confidence and identity centrality.

	**Epistemic confidence**	**Identity centrality**
Formation conditions	Constrained by cognition of evidence	Social opportunity (Stark and Finke, 2000), voluntary choice (Kierkegaard, 1843/1985; Luhrmann, 2012).
Extinction conditions	Cognition of contrary evidence	Value conflict with group leaders or group in general (Sauvayre, 2011; Bialecki, 2017).
Action output	Decision theoretic, instrumental	Symbolic, experiential (Van Leeuwen and Van Elk, 2019; Luhrmann, 2020), solidarity building (Sosis and Alcorta, 2003; Bulbulia, 2004, 2012; Alcorta and Sosis, 2005; Henrich, 2009).

Much more can be said about each of these dimensions of variation. But the broad outlines are clear: Epistemic confidence is, with various exceptions, likely to respond to evidence and guide instrumentally rational actions; identity centrality is likely to respond to social pressures and guide in-group-oriented behavior and self-presentation. So this is a distinction that makes a host of differences.

With regards to normative philosophical research, it is fair to say that this distinction raises a range of questions. The most basic one is this: Should the norms of evidence and truth that seem clearly to apply to epistemic confidence transfer over to identity centrality? In point of fact, it seems that identity centrality *is* far less constrained by evidence. But *should* it be? This is an important question, whose answer I don't know, that the present work at least puts us in a position to ask more clearly. And if it turns out that the proper norms for the respective psychological features do differ, it is even more important to develop measurement tools that would detect which of the two phenomena we are dealing with for any given “belief” set. Otherwise we wouldn't know which norms are applicable in any given case.

One of the reasons, I suspect, why such tools are lacking is that teasing out the distinction using survey instruments is likely to be extremely hard. That is why the difference is easier to notice in ethnographies, which incorporate observation of non-verbal behavior and of more nuanced verbal behavior.

Yet building measurement scales would still be worth the attempt, and I suggest that the above chart could be used to generate proxies for the features in question in relation to specific belief contents. To what extent is one's belief (say) that God is triune constrained by evidence vs. being voluntarily chosen? To what extent is it likely to be rejected due to contrary evidence vs. value conflict with group leaders? Does it guide instrumental or symbolic actions? No doubt any such scale would elicit some noise in addition to signal. Nevertheless, appropriate measurement tools could well be crafted that get at important and striking differences in the ways people can and do have “strong” beliefs.

## Author Contributions

The author confirms being the sole contributor of this work and has approved it for publication.

## Funding

This article is funded by Dr. Rüdiger Seitz, *via* the Volkswagen Foundation, Siemens Healthineers, and the Betz Foundation.

## Conflict of Interest

The author declares that the research was conducted in the absence of any commercial or financial relationships that could be construed as a potential conflict of interest.

## Publisher's Note

All claims expressed in this article are solely those of the authors and do not necessarily represent those of their affiliated organizations, or those of the publisher, the editors and the reviewers. Any product that may be evaluated in this article, or claim that may be made by its manufacturer, is not guaranteed or endorsed by the publisher.

## Footnotes

^1^The Vineyard is now an international movement, but it originated in the US and still has many features characteristic of American Evangelicalism.

^2^This characterization of “knowledge” is intended for purposes of this definition only and not as a fundamental analysis of that term. Note also that I am not saying that an epistemically confident belief state must *be* knowledge—only that the person who has it must *feel* (to some degree) like it approximates knowledge.

^3^The in-group in Johan's case would be socially liberal South Africans. I should also note that there are interesting relations between the two kinds of “strength.” In particular, one uses one's perceived knowledge about one's group to figure out what “beliefs” should be accorded identity centrality. So epistemic confidence does play a foundational role in relation to identity centrality, even if identity centrality guides symbolic behaviors that often seem to go against it. This is similar to the anti-symmetric cognitive governance relation I discuss in my (2014) paper, and I thank one anonymous referee for raising the issue.

^4^I focus here on psychology of religion, but my points are likely to generalize to other sub-fields as well.

^5^See also Van Leeuwen, 2014, 2017a,b, 2018 for parallel comparisons between factual belief, which has epistemic confidence, and religious credence, which has identity centrality.
